# Arabo-Persian Perspective on Classification of Psychotic Disorders

**DOI:** 10.1192/bjo.2024.246

**Published:** 2024-08-01

**Authors:** Homayun Shahpesandy

**Affiliations:** Sherwood Hospital, Nottingham, United Kingdom

## Abstract

**Aims:**

The first medical textbook in Arabic, ‘Firdaus as-Hikmah’ (The Paradise of Wisdom) by Tabari (808–861) was composed in in year 848. Tabari's classification of insanity is simple and in term of psychosis, he talks about syndromes of ‘*Hearing voices in the head’* (hallucinatory psychosis), ‘*humm-al-hubbi’* (love fever) and ‘*humm-al-sehr’* (fever from enchantment). The first classification of ‘junun’ (psychosis) comes from Râzī (854–925), who in his ‘*Al-Hāwī fil Tib’* (The System of Medicine) divides ‘insanity’ (psychosis) into ‘al-junun al-thābet’ or ‘permanent madness’, and ‘*a'rāz tābea-tu leamrāz’* or ‘symptomatic psychotic disorders'. The first medical textbook in Persian language*, ‘Dāneshnāma’* (Medical Encyclopaedia) by Hakim Maysarī, completed in 978–9 mentions only melancholia and ‘rejā’ (pseudocyesis/pseudopregnancy) and no other psychotic conditions. Prospective generations of Arabic-inscribing physicians, including Majūsī, also known as Haly Abbas (949–990), Avicenna (980–1037), and Persian-inscribing physicians such as Bokhârī̄ (? −983) and Jorjânī̄ (1040–1137) are strongly influenced by Râzī̄ and use similar taxonomy of psychotic disorders. Moreover, the taxonomy introduced by Râzī̄ and other mediaeval physicians has been used in Arabic and Persian speaking medical communities until the past century. Nevertheless, these were substituted by Latin-based language vocabulary reflecting the International Classification of Diseases (ICD).

The aim of this work is to review the input of Arabic and Persian schools in the development of psychiatric knowledge and classification.

**Methods:**

Literature search of ‘Firdaus-al-Hikmah’ of Tabari, ‘*Kitāb al-Hāwī fī al-ṭibb*’ of Râzī̄, ‘*Kitābu'l Malikī’* (The Royal Book) by Majūsī, ‘*Al-Qānūn fī al-Ṭibb’* (Canon of Medicine) of Avicenna in Arabic; and ‘*Hidâyat al-Mutaʽallemin fi al-Ṭibb*’ (A Guide for Medical Students) of Al-Akhwayani Bokhârī and *'Zakhīra-i Khwârazmshâhī'* (The Treasure of Kwārazmshāh) and *Al- ‘Aghrād'ul tibiyah wa'al-mabāhith'ul Ala'iyah’* (The Aims of Medicin) of Jorjânī in Farsi.

**Results:**

**1. ‘Transient‘ or symptomatic psychotic disorders, resulting from direct or indirect brain damage:**

1. ‘*Ekhtelāt-ul-takhayyol’* (disorder of perception), ‘when patients imagine perceptible things, such as seeing people, hearing sounds, or sensing smells that have no external reality’.
2. *'Ekhtelāt-al-fekr’* (thought disorder), when the perception is intact and patients perceive the outside reality as it is, however, their thinking is impaired.
3. ‘Ekhtelāt-al-aqhl’ ('corruption of the mind’), or ‘junun (madness), defined as a condition when patients say things they should not say, like things they should not like, wish unreasonable things, demand what is not demanded, do things they should not do, or hate things that they normally do not hate.
4. '*Sobārā'*, portrayed as a form of agitated madness resulting from ‘sarsām’ (meningitis/encephalitis).

**2. 'Permanent’ psychotic disorders also considered as primary ‘brain’ diseases:**

1. Mania, described as the worst kind of insanity, presenting symptoms of paranoia, constant anxiety, agitation, hyperactivity, vindictiveness, insomnia, hostility, and ferocity.
2. *‘Dâ-al-kalb'* (‘dog's disease'), portrayed as a mixed psychosis with a fluctuating picture of anger and playfulness, as well as hostility mixed with gentleness.
3. '*Qutrub*', outlined as a psychosis when affected individuals dislike people's company and run away from society, rarely resting, and aimlessly moving as if they were in fear of running from someone. Patients become forgetful, and their behaviours disorganised.

**Conclusion:**

The Arabo-Persian classification of mental disorder was progressive and generated a common nomenclature in the Arabo-Persian speaking medical communities, serving the mutual understanding of experts. Moreover, the taxonomy developed was relatively precise and stable, corresponding to modern classification systems. Psychoses were categorised into ‘transient’ and ‘permanent’ disorders, which were considered as a primary ‘brain disease’ of multifactorial aetiology, a concept introduced by Griesinger in the 19th century, known as the ‘organic model’ of mental illnesses.

